# Quantitative susceptibility map from the patients with chronic heart failure

**DOI:** 10.1186/1532-429X-18-S1-P14

**Published:** 2016-01-27

**Authors:** Jiun-Jie Wang, Gigin Lin, Chao-Hung Wang, Yu-Hsiang Juan, Pei-Ching Huang

**Affiliations:** 1 Medical Imaging and Radiological Sciences, ChangGung University, TaoYuan county, Taiwan; 2 Medical Imaging and Intervention, ChangGung Memorial Hospital, TaoYuan county, Taiwan; 3 grid.454209.e0000000406392551ChangGung Memorial Hospital, Keelung, KeeLung Taiwan

## Background

Iron deficiency can be related to patients with chronic heart failure. Although T2* mapping by MRI could be used as a non-invasive interrogator for the assessment of the iron loading, unfortunately the result is only with limited success. Quantitative susceptibility mapping is a further development using susceptibility weighted image. Previous studies suggested a strong linear co-relation between the iron concentration and the magnetic susceptibility. Therefore we used this technique to determine the iron loading and the measured susceptibility from the heart of patients with chronic heart failure.

## Methods

50 patients with chronic heart failure were recruited from the local community. The study was approved by the institutional review board and is complied with the declaration of Helsinki. Informed consent was acquired from all participants. MR images were acquired using a 3 T scanner (Skyra, Siemensm Erlangen Germany). 3D fully flow compensated T2* weighted images were acquired with multiple TE of 2.21;4.18;6.15;8.12;10.09;12.06;14.3 and 16 msec. Additional imaging parameters included TR(844.8 msec), Field of View (332 mm by 379 mm) and matrix size (118 by 224). The quantitative susceptibility map was calculated by using software downloaded from Cornell MRI research Lab. Both the susceptibility weighted images and the T2 * map were calculated for comparison.

## Results

Patients were divided into 2 groups by the left ventricle ejection fraction. The result showed that patients with the left ventricle ejection fraction smaller than 50%, both the T2* and the quantitative susceptibility map were significantly smaller, when compared to patients with a larger left ventricle ejection fraction. The increased iron loading can be related to the cardiac function of the patients. Because T2* could vary from scanner to scanner, it is expected that quantitative susceptibility map can be a potentially useful image based biomarker for the early prognosis of chronic heart failure.

## Conclusions

The quantitative susceptibility mapping in the diseased heart is feasible. It provides quantitative estimation of the iron loading and therefore can be of great interest in the studies related to iron deficiency in chronic heart failure.

